# Exploring the Potential of Vine Shoots as a Source of Valuable Extracts and Stable Lignin Nanoparticles for Multiple Applications

**DOI:** 10.3390/ijms24065165

**Published:** 2023-03-08

**Authors:** Ana Rita Pereira, Carina Costa, Nuno Mateus, Victor de Freitas, Alírio Rodrigues, Joana Oliveira

**Affiliations:** 1 Laboratório Associado para a Química Verde–REQUIMTE, Departamento de Química e Bioquímica, Faculdade de Ciências, Universidade do Porto, Rua do Campo Alegre, 687, 4169-007 Porto, Portugal; 2 LSRE-LCM-Laboratory of Separation and Reaction Engineering-Laboratory of Catalysis and Materials, Faculty of Engineering, University of Porto, Rua Dr. Roberto Frias, 4200-465 Porto, Portugal; 3 ALiCE-Associate Laboratory in Chemical Engineering, Faculty of Engineering, University of Porto, Rua Dr. Roberto Frias, 4200-465 Porto, Portugal

**Keywords:** lignin, dialysis, phenolic compounds, antioxidant, nanoparticles

## Abstract

Large amounts of vine shoots are generated every year during vine pruning. This residue still presents many of the compounds found in the original plant, including low molecular weight phenolic compounds and structural compounds such as cellulose, hemicellulose, and lignin. For wine-producing regions, the challenge is to develop alternatives that will increase the value of this residue. This work proposes the full valorization of vine shoots, focusing on the extraction of lignin by mild acidolysis for the preparation of nanoparticles. The effect of the pretreatment solvents (ethanol/toluene, E/T, and water/ethanol, W/E), on the chemical and structural features of lignin, was evaluated. The chemical analysis suggests similar composition and structure regardless of the pretreatment solvent, although lignin isolated after pretreatment of biomass with E/T showed a higher content of proanthocyanidins (11%) compared with W/E (5%). Lignin nanoparticles (LNPs) presented an average size ranging from 130–200 nm and showed good stability for 30 days. Lignin and LNPs showed excellent antioxidant properties (half maximal inhibitory concentration, IC_50_ 0.016–0.031 mg/mL) when compared to commercial antioxidants. In addition, extracts resulting from biomass pretreatment showed antioxidant activity, with W/E presenting a lower IC_50_ (0.170 mg/mL) than E/T (0.270 mg/mL), correlated with the higher polyphenol content of W/E, with (+)-catechin and (−)-epicatechin being the main compounds detected. Overall, this work shows that the pre-treatment of vine shoots with green solvents can yield (i) the production of high-purity lignin samples with antioxidant properties and (ii) phenolic-rich extracts, promoting the integral reuse of this byproduct and contributing to sustainability.

## 1. Introduction

The European Union (EU) produced 165 million liters of wine in 2020, and by this measure, the wine industry is one of the most important food industries in the world. According to the International Organization of Vine and Wine [1], Portugal ranks 11^th^ among major wine producers and exporters worldwide. With this, every year, millions of tons of organic waste (grape pomace, grape stems, lees, and vine pruning material), wastewater, and inorganic residue are produced, which contributes to significantly increasing greenhouse gas emissions. For instance, it has been estimated that the Portuguese wine industry generates between 1.2 and 3.5 t/ha of waste or co-products annually during the vintage season [2]. In 2019, around 3.4 × 10^5^, 5.8 × 10^6^, and 1.3 × 10^7^ t of vine shoots were produced in Portugal, Europe, and the world, respectively [3]. Due to their high calorific value, these residues are typically left in the vineyard or used as domestic fuel, resulting in the release of greenhouse gases such as N_2_O, CO, CH_4_, and NO_2_ into the atmosphere [4,5]. To reduce the impact of these co-products on the environment and increase the value of the winemaking process, new green strategies to reuse, recycle, and recover these residues—especially organic residues—are constantly being developed. Vine shoots are a complex matrix with structural components such as cellulose (32–40%), hemicellulose (5–27%), and lignin (16–39%) [6,7,8], with other minor compounds such as lipids, vitamins, and more simple phenolic compounds. Due to their antioxidant properties and beneficial effects on human health, including cardioprotective, anticancer, anti-inflammation, antiaging, and antimicrobial properties [9], phenolic compounds have received the most attention, particularly from the industry [10,11]. It has been suggested that vine shoots could be used as a source of sugars that could be turned into bioethanol, xylitol, lactic acid, and biosurfactants [12,13]. In addition, it has been investigated whether this co-product could be applied in the production of various materials such as particle boards, biochar, and paper [14,15,16,17].

Lignin is a natural aromatic biopolymer that can be used as a raw material in various settings. It is inexpensive, stable, relatively nontoxic, and highly biodegradable. Several methods have been developed in the industrial sector to convert lignin directly into heat or electricity, bio-oil, or combustible biogas [18]. Due to its complex and varied structure and low solubility, lignin has been used in a number of little-known applications, including surfactants, adhesives, and dispersants. Drug delivery vehicles, scaffolds for tissue engineering, materials for dressing wounds, lignin-based nanoparticles (NPs), and hydrogels are just a few examples of the biomedical uses for lignin composites [19]. Natural lignocellulosic fibers from agricultural crops have found applications in many industries due to their low cost, lightweight, superior mechanical properties, and abundance [20,21]. Furthermore, since they are eco-friendly, non-toxic, and biodegradable, natural fiber-reinforced polymer composite materials have largely replaced synthetic carbon-based materials, particularly in plastics, electronics, packaging [22], and the automotive industry [23]. There are three fundamental phenylpropanoid monolignols in the composition of lignin: sinapyl, coniferyl, and *p*-coumaryl alcohols. These subunits are the precursors of the main moieties of lignin structure: syringyl (S), guaiacyl (G), and *p*-hydroxyphenyl (H), which differ in the methoxylation of the aromatic units. Moreover, the structural units of lignin can comprise several functional groups. The most frequent ones are primary and secondary aliphatic hydroxyl, aromatic methoxyl and phenolic hydroxyl, and in minor amounts, the carbonyl (aldehydes and ketones), and carboxyl groups [24]. The plant species (softwood, hardwood, or grass), the morphological part of the plant, and the delignification process determine the structural characteristics (functional groups and type of linkage) and composition of lignin [25,26]. It is essential to select a process that has a low impact on native lignin structure, such as dioxane and “milled-wood” lignins in order to avoid extensive lignin structural changes caused by the isolation required for the characterization of lignocellulosic biomass [27]. The extraction and structural characterization of lignin from various herbaceous plants, such as rice [28], cotton, corn stalks and roots, sugarcane, and tobacco, have been described in the literature [27]. In the first case, the authors used ultrasound-assisted extraction in an alkaline medium to promote the extraction of lignin and cellulose from rice straw, and their yields increased with sonication time from 10 to 30 min [28]. Esteves et al. [27] used a mild acidolysis to extract lignin from two morphological parts (stalks and roots) of different sources for the production of added-value products, such as vanillin and syringaldehyde, in the perspective of a biorefinery. Prozil and coworkers [29] reported the extraction of lignin from grape stalks using a modified acidolytic procedure with various steps and organic solvents.

Most of the works concerning the valorization of by-products focused on one class of compounds, overlooking the others. This work proposes the total extraction of bioactive and structural compounds (lignin), aiming for the full valorization of vine shoot residues. For that, a mild acidolysis methodology was used to obtain lignin (with little structural modifications) that was used to produce stable nanoparticles with the potential to be used in different technological applications (food, cosmetic, and pharmaceutical industries). Moreover, as a result of biomass pretreatment, phenolic compound-rich extracts were obtained with important antioxidant properties, contributing to the bioeconomy and sustainability.

## 2. Results and Discussion

### 2.1. Characterization of the Extracts Resulting from the Pretreatment Process

**Scheme 1:**
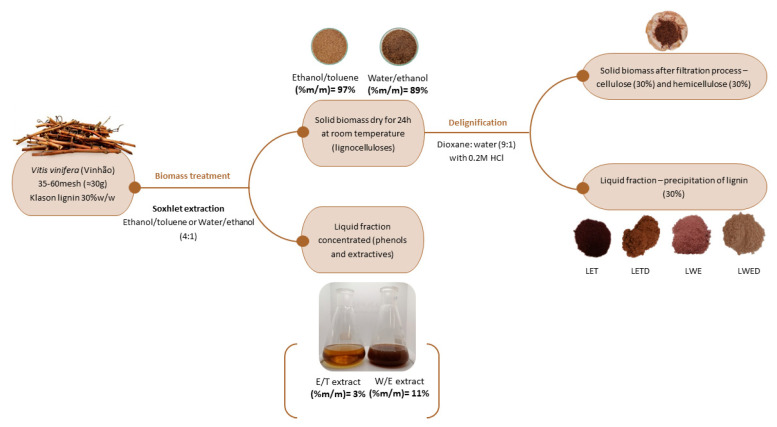
Lignin content in the vine shoots and the yield of liquids and solids recovered after each procedure.

The biomass was pretreated prior to the delignification of vine shoots by Soxhlet extraction using ethanol/toluene (E/T) or water/ethanol (W/E), and the lignin was then isolated from the dried biomass by mild acidolysis (Scheme 1). The chemical composition analysis of vine shoots revealed that this material contained low amounts of inorganic compounds and other extractives (2.5% and 4.9%, respectively). Non-cellulosic carbohydrates make up 21% of the weight of the biomass, and the lignin content (Klason lignin) is about 30%. These numbers are in line with those that have been reported in the literature [6,16]. The yield of lignin isolation from the mild acidolysis procedure was about 30%, calculated with reference to the total lignin content (Klason and soluble lignin) determined in vine shoot samples.

Compared to the 3% obtained for the E/T extract, the W/E extract accounts for approximately 11% of the initial biomass. This suggests that this solvent mixture has been more effective at removing the phenolic and other bioactive compounds from biomass, as previously documented in the literature [30,31]. When compared to the E/T extract, which had a total polyphenol (TP) of 82.8 ± 0.7 mg GAE (gallic acid equivalents)/g extract, the Folin–Ciocalteu method revealed a much higher value (*p* < 0.001) of TP content (328.9 ± 0.4 mg GAE/g extract). The antioxidant activity of W/E extract showed a similar pattern, with a lower IC_50_ value (IC_50_ 0.170 mg/mL) in comparison to a higher value for E/T extract (IC_50_ 0.270 mg/mL). This is a consequence of the higher phenolic content of this extract.

#### 2.1.1. Proanthocyanidins

The phenolic compound composition of extracts W/E and E/T was determined by mass spectrometry coupled to diode array detection and mass spectrometry (LC-DAD/MS). The isomer monomeric units (+)-catechin (*m*/*z* 289) and (−)-epicatechin (*m*/*z* 289) were the main flavan-3-ols detected in both samples. Moreover, three B-type procyanidin dimers (*m*/*z* 577) were also identified in both extracts. The unequivocal identification of procyanidins (PAs) was achieved by their characteristic fragmentation (MS^2^) patterns that include water loss (MS^2^ 407and 559), heterolytic ring fission (HRF, MS^2^ 451), quinone methide (QM, MS^2^ 289) fission, and the retro-Diels-Alder (RDA, MS^2^ 425) [32,33]. Gallic acid esters of flavan-3-ol and PAS dimers were also identified: (epi)catechin-(epi)catechin gallate (*m*/*z* 729) and a 3-methyl epigallocatechin gallate (*m*/*z* 471). The first form produced ions (MS^2^) at *m*/*z* 577, 559, and 407, which are typical fragments of B-type procyanidin dimers. The gallic acid ester position is fundamentally based on QM fission. Thus, the ions at *m*/*z* 441 were crucial in assigning the gallic acid ester to the bottom positions. Phenolic compounds detected in these samples were already reported in the literature to be present in vine shoots, being present in higher amounts in the WE extract [30,31].

#### 2.1.2. Sugars

The analysis of the sugar content of both extracts showed that samples are mainly composed of glucose, with residual amounts of fructose and sucrose. As expected, the W/E extract resulting from Soxhlet extraction with water/ethanol (dark brown) exhibited a higher sugar content (681.3 ± 8.8 mg glucose/g extract) than the extract from the biomass treatment with ethanol/toluene (112.6 ± 7.6 mg glucose/g extract). The treatment with water allowed a more effective removal of sugars present in the biomass, which means that the isolated lignin will present a lower amount of this component. In this way, a sugar-rich extract is obtained using a combination of environmentally friendly solvents in the treatment of vine shoots. In other works, Jesus and coworkers [12] also detected the presence of xylose and arabinose in liquid fractions resulting from a sequential autohydrolysis treatment at 180 °C for 60 min of vine pruning residues. Then, bioethanol was produced by separate and simultaneous saccharification and fermentation. Rivas et al. [7] added the presence of galactose (2.5 ± 0.3 wt% in oven-dry basis) and mannose (1.0 ± 0.1 wt% in oven-dry basis) after the treatment of this biomass with aqueous solvent extraction at 130 °C. Independent of the biomass treatment or extraction method used, these results show that vine shoots is a potential renewable source of sugars to produce value-added products for different industries.

### 2.2. Cellulose and Hemicellulose Fraction

Solid fractions containing 30% cellulose and 30% hemicellulose were produced following the delignification process. These percentages are consistent with the literature’s descriptions, and depending on the extraction technique, different levels of polysaccharides may be obtained [7]. Additionally, a variety of bioproducts can be produced by individually valorizing these fractions. The literature describes various fractionation techniques based on one-step fractionation with immiscible solvents, with 1-butanol (green solvent) being the solvent that allowed more favorable results [34]. After fermentation, cellulose solids go through enzymatic hydrolysis to produce glucose solutions, which can be used as fermentation media to produce bioproducts such as bioethanol or biofuels [35]. For hemicellulose, when exposed to acidic conditions, pentosan polysaccharides are converted into pentoses (xylose and arabinose) by dehydration, leading to the formation of furfural, whose excellent yields make this procedure a source of commercial production [36]. In the same way, hexoses (such as glucose, galactose, or mannose) can be produced by hydrolyzing hexosans and then dehydrated to produce hydroxymethylfurfural. Furfural and hydroxymethylfurfural are significant platform chemicals that can be used to produce a variety of fuels and bioproducts [36,37]. The majority of the byproducts produced during partial hydrolysis of hemicelluloses are polysaccharide fragments (low molecular polysaccharides and/or oligosaccharides). Oligosaccharides can be utilized as prebiotics, for instance, in the food and pharmaceutical industries due to their bioactivity [38].

### 2.3. Characterization of Lignin from Vine Shoots Using Mild Acidolysis Extraction

#### 2.3.1. Composition of the Isolated Lignin Samples

The chemical composition of isolated lignins is depicted in Table 1. For the isolated lignins, a maximum of 1.2% of inorganic content was found, while the content of co-extracted carbohydrates was between 4.2 and 6.9%, accounting for a maximum of 7.2% of the referred contaminants in the lignin pretreated with water/ethanol (LWE) sample.

One of the main objectives of this work was to evaluate the influence of the solvent mixture used in the Soxhlet extraction (pre-treatment of the vine shoots) and the purification of the sample by dialysis in the final structure and composition of lignin. Based on the previous results, there are no significant differences in the composition of the different lignin samples, considering the inorganic material and the carbohydrates. It is also important to note that mild acidolysis gives origin to lignin with a low content of contaminants.

#### 2.3.2. Proanthocyanidins Content in Lignin Samples

According to the literature, the lignocellulosic biomass from the wine industry co-products presents a considerable percentage of proanthocyanins in its composition. It is described that grape stalks can have approximately 6% of tannins [39,40] although in some cases, their amount can reach ≈17% [8]. Considering that vine shoots are derived from the same plant, the evaluation of the presence of these compounds is essential. The undefined and complex structure of lignin and/or its possible interaction with tannins was suggested to be the reason for the extremely difficult delignification of this type of biomass. Table 1 shows that samples LET and LETD present a higher percentage of proanthocyanidins with values around 11 and 9%, respectively, when compared with the 5 and 3% of proanthocyanidins that were obtained for LWE and LWED, respectively. This means that the pre-treatment with water/ethanol was more efficient in the elimination of tannins from the lignocellulosic biomass. The resulting liquor from this extractive process had a more intense brown color than the liquor obtained using ethanol/toluene. The literature states that water alone or water mixed with other solvents such as methanol, ethanol, acetone, or NaOH can effectively extract lignin free of tannins. The ethanol-toluene mixture is better suited to remove waxes, fats, resins, and wood gums [41].

#### 2.3.3. Molecular Weight and Polydispersity of Lignin Samples

The molecular weight (Mw) and polydispersity (Mw/Mn) of the isolated lignins were determined by gel permeation chromatography (GPC). The values obtained are between 43,000–49,000 g/mol (Table S1). The lignins resulting from the vine shoots pre-treated with ethanol/toluene (LET and LETD) have a higher molecular weight of 45,243 and 48,249, respectively, when compared with the lignins resulting from the vine shoot pre-treatment with water/ethanol (LWE: 43,340 and LWED: 47,254). Moreover, the lignin subjected to dialysis shows a higher molecular weight than the correspondent lignins that were not submitted to this purification step.

#### 2.3.4. Quantitative Carbon-^13^ Nuclear Magnetic Resonance (^13^C NMR)

The quantitative ^13^C NMR spectra of the analyzed lignins, with the main assignments identified, are presented in Figure S1. The assignments and quantification of each structure, linkage, and functional groups identified in the lignin structure were made based on reference spectra and data available in the literature [27]. The ^13^C NMR results, presented in Table 2, are reported as the ratio of the integral of a given carbon signal to one-sixth of the integral of aromatic carbons (Ar).

The quantitative data from ^13^C NMR enables the determination of the main structural characteristics of isolated lignins (Table S2). The results confirm that lignin isolated by mild acidolysis after pre-treatment of the vine shoots with water/ethanol and purified by dialysis (LWED) has the highest content of non-condensed structures (β-*O*-4 structures, 65 per 100 aryl units) and the highest condensation degree. It was observed that the condensation degree increases in the following order: LET (9%) < LETD (13%) < LWE (19%) < LWED (21%). Moreover, there are no differences in the number of the main structural units, S, G, and H, between all the lignins, confirming that the dialysis and the Soxhlet extraction do not have an effect on lignin structure, resulting in lignin with a structure closer to the native one.

#### 2.3.5. Phosphorus-^31^ Nuclear Magnetic Resonance (^31^P NMR)

Lignin’s properties are influenced by the proportion of aliphatic and phenolic hydroxyl groups in its structure. In order to elucidate the amount and type of hydroxyl groups present in these samples, isolated lignins (LETD and LWED) were phosphitylated with 2-chloro-4,4,5,5-tetramethyl-1,3,2-dioxaphospholane and analyzed by quantitative ^31^P NMR. The ^31^P NMR spectra of the phosphitylated lignins and the respective quantification of the different hydroxyl groups are described in Supplementary Materials (Figure S2 and Table S3, respectively). Aliphatic hydroxyl groups are associated with signals between 149 and 146 ppm, while phenolic hydroxyl groups are associated with signals between 143 and 137 ppm. The content of phenolic OH in the lignin structure was 1.01 mmol/g of lignin for sample LETD and 0.84 mmol/g of lignin for sample LWED. In the phenolic hydroxyl region, the signals in the range of 143.1–142.5, 140.2 to 139.4, and 138.2 to 137.8 ppm are assigned to hydroxyl groups in non-condensed guaiacyl, syringyl, and *p*-hydroxyphenyl units, respectively. The presence of these signals in isolated lignin fractions suggests GSH-type lignin, which agrees with the results obtained from FTIR. Relative to the higher quantity of aliphatic hydroxyls (3.20 mmol/g lignin for LETD and 2.80 mmol/g lignin for LWED), these could be overestimated values once the residual carbohydrates present in the samples can undergo phosphorylation of the hydroxyl groups that are quantified by ^31^P NMR as aliphatic OH groups, as described in the literature [27]. In this sense, the aliphatic hydroxyl content was not considered for the total phenolic hydroxyls.

#### 2.3.6. Fourier-Transform Infrared Spectroscopy (FTIR)

**Figure 1:**
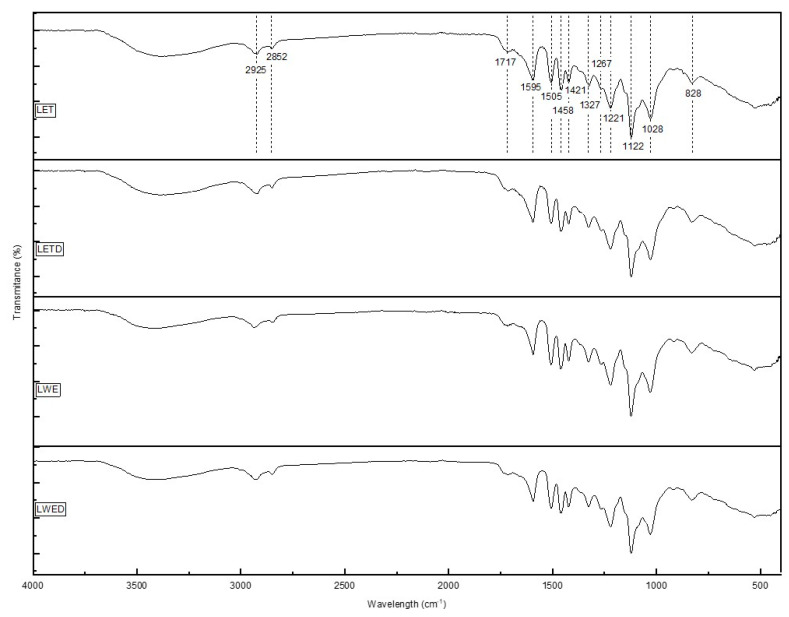
FTIR absorption spectra for isolated lignins LET, LETD, LWE, and LWED.

Figure 1 displays the FTIR spectra of the different lignin samples. As can be observed, the spectra are similar for all lignin samples, with the same absorption bands and equivalent intensities.

Lignins have characteristic signals in the region between 700 and 1600 cm^−1^, and typical chemical group stretching was observed between 2800 and 3500 cm^−1^. Characteristic bands of the lignin structure present at 1593, 1505, and 1422 cm^−1^ were assigned to the aromatic skeletal vibration. The presence of the three basic units of lignin was also evidenced, where the syringyl (S) ring absorption is observed at 1327 cm^−1^, the guaiacyl (G) ring appears with a small shoulder at 1267 cm^−1^, and for *p*-hydroxyphenyl (H) units, an absorption band is observed at 833 cm^−1^. The presence of both absorption bands at 1267 and 833 cm^−1^ and a dominating peak at 1121 cm^−1^ are typical of S:G:H lignins [42,43,44]. The absorption band at 1717 cm^−1^ in lignin indicates the presence of unconjugated C=O stretching (vibration of unconjugated ketones, ester, or carboxylic groups). The FTIR spectra also showed two peaks with low intensity at 2852 and 2925 cm^−1^, which can be attributed to symmetric and asymmetric C-H stretching in the aromatic methoxyl group and the methyl and methylene groups of the side chain of lignin. On the other hand, according to the literature, these absorption bands also appear in the FTIR spectrum of proanthocyanidins [45,46], causing a noticeable interference in all samples; even small amounts can contribute to the intensity of these peaks. Asymmetric C-H deformation in -CH_2_- and -CH_3_- groups can be found at 1461 cm^−1^. The band at 1221 cm^−1^ is attributed to C-C plus C-O plus C=O stretch, whereby G condensed > G esterified [42,43,44], and the band at 1028 cm^−1^ is associated with C-H (in-plane) deformation of the guaiacyl ring plus deformation of primary C-O and in ether with contribution from the stretching of unconjugated C=O [44].

### 2.4. Lignin Nanoparticles—Size, Polydispersity Index, and Zeta-Potential

Attending to the increasing growth of lignin used in biological applications, such as nanoparticles and hydrogels, the four lignin samples (LET, LETD, LWE, and LWED) were used to produce lignin nanoparticles (LNPs) using nanoprecipitation or dialysis, and their physical-chemical properties were evaluated over time. By comparing the size and the polydispersity index of LNPs produced by nanoprecipitation and dialysis, the results show significant differences (*p* < 0.001) in the size of the LNPs produced by nanoprecipitation (120–200 nm) when compared with the ones obtained by dialysis (250–505 nm). A similar trend was observed for the polydispersity index (0.19–0.26 compared with 0.37–0.44, respectively). LET and LWE lignin samples yielded nanoparticles with larger sizes and polydispersity index when compared with the ones observed for LNPs obtained from LETD and LWED (Figure S3).

**Figure 2:**
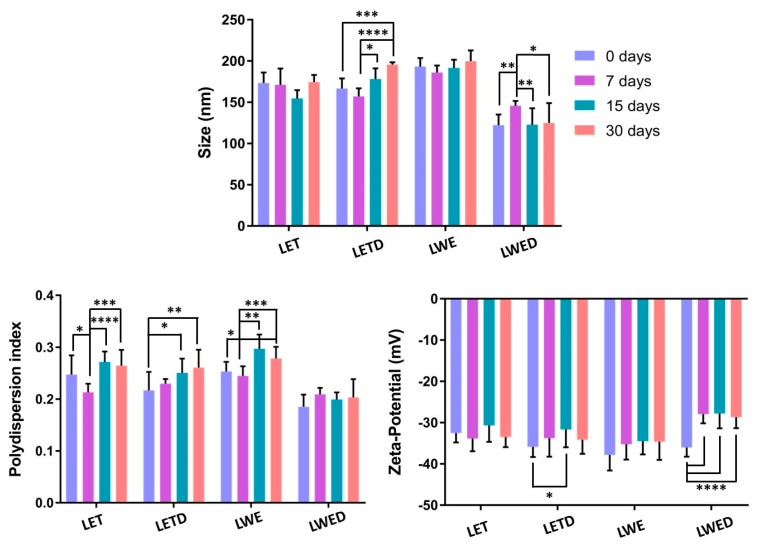
Evaluation of LNPs stability for 30 days for their size, polydisperse index, and zeta-potential. Statistical analysis was performed by ANOVA, followed by Sidak’s multiple comparison tests. Different asterisks indicate a statistical difference: *p* < 0.05 (*), *p* < 0.005 (**), *p* < 0.001 (***) and *p* < 0.0001 (****) Error bars represent the mean ± s.d (n = 9).

Bearing all this, the stability of the LNPs prepared by nanoprecipitation was monitored over time. In a brief comparison between the different lignin samples (Figure S4), the results showed significant differences, especially in the particle size, with LNPs produced with LWED yielding lower values (LET: 173.3 ± 4.3 nm; LETD: 166.7 ± 4.0 nm; LWE: 193.3 ± 3.4 nm and LWED: 122.1 ± 4.3 nm). Depending on the target application, all samples present appropriate sizes as LNPs. Relatively to the polydispersity index, the values ranged between 0.1–0.4, which means that the samples are moderately dispersed. A similar trend was observed for the polydispersity index with LNPs prepared with LWED, showing lower values (0.18 ± 0.02) when compared with the other lignin samples: LET (0.24 ± 0.04), LETD (0.22 ± 0.04), and LWT (0.25 ± 0.02). The zeta-potential was below −30 mV for all samples, which suggests a good physical-chemical stability of the nanosuspension due to the electrostatic repulsion of individual particles. In general, LNPs were revealed to be stable for 30 days (Figure 2). When compared to the particle size on the first day, the size of the LETD increased to values of about 200 nm after 30 days. The results in the subsequent lignin samples remained remarkably close to those observed on the first day, with the exception of the LWED sample, in which a greater change in the polydispersion index values was observed, although it was below 0.4. The nanoparticles kept their negative charge on the surface in all samples, with the LWED sample experiencing a slight reduction to values below −30 mV after 7 days. In this sense, lignin from vine shoots demonstrated its potential for use in the production of stable LNPs.

### 2.5. Antioxidant Activity of Soluble Lignin and Lignin Nanoparticles

The chemical structure of a molecule directly affects its antioxidant capacity. In this work, the antioxidant activity of lignin and LNPs were evaluated using a common colorimetric assay based on the elimination of the π-radical. Although there may be several reaction mechanisms for the scavenging, many radical scavengers react with DPPH (2,2-diphenyl-1-picrylhydrazyl) by giving one electron and one proton. The results of the antioxidant activity of lignin solutions and respective nanoparticles (Table 3) were expressed in terms of IC_50,_ which correspond to the amount of the antioxidant required to reduce the initial concentration of the DPPH% radical by 50%, and in terms of the radical scavenging index (RSI), which is the inverse of IC_50_. This means that the lower the IC_50_ of an antioxidant, the higher its antioxidant efficiency and RSI value.

DPPH results showed a clear correlation between lignin concentration and antioxidant activity, increasing the inhibitory effect at higher concentrations for all samples. It was observed that the antioxidant activity of soluble lignin and LNPs was higher than some commercial antioxidants, such as BHT (butylated hydroxytoluene) and BHA (butylated hydroxyanisole), whereas it was lower than trolox, gallic, and ascorbic acids. The highest antioxidant capacity demonstrated to the soluble LET and LETD could be attributed to the presence of the highest phenolic content and the lowest carboxylic acid content previously quantified by the ^31^P NMR, which is described in the literature as factors that affect the antioxidant behavior of lignins [47,48,49]. Impurities such as carbohydrates can also negatively affect the antioxidant activity since the formation of hydrogen bonding with lignin blocks the formation of free radicals [50]. In fact, the soluble lignin with higher carbohydrate content (around 6.9%) in the LWE sample showed the lower antioxidant capability. By comparing the IC_50_ and RSI values obtained for lignin in solution, similar results for the antioxidant capacity of different types of lignins were found by other authors [51,52]. In general, the LNPs showed lower IC_50_ values when compared to the respective soluble lignin. However, it is important to highlight that LNPs were dispersed in water to avoid disintegration while the reference antioxidants and soluble lignins were dispersed in 50% (*v*/*v*) of ethanol, which may influence the obtained results. Concluding, the high antioxidant activity of the lignin samples suggests that they could act as potential antioxidants for the food, cosmetic, and pharmaceutical industries instead of using BHT.

**Table 3:** IC_50_ values of the lignin and nanoparticles from vine shoots were obtained in the current study with other antioxidants described in the literature. For comparison, RSI values were computed (=1/EC_50_).

Sample	IC_50_ (mg/mL)	RSI (1/IC_50_)
LET (soluble lignin)	0.017	59
LETD (soluble lignin)	0.016	63
LWE (soluble lignin)	0.030	33
LWED (soluble lignin)	0.026	38
LET nanoparticles	0.031	32
LETD nanoparticles	0.029	34
LWE nanoparticles	0.029	34
LWED nanoparticles	0.030	33
Commercial antioxidants		
Gallic acid	0.002 (in this work)	500
Trolox	0.003 (in this work)	333
Ascorbic acid	0.005 (in this work)	200
Vitamin E	0.0037 [47]	263
BHT	0.038 [52]	26
BHA	0.056 [52]	18

## 3. Materials and Methods

### 3.1. Preparation of Vine Shoots

First, 250 g of vine shoots from the Vinhão variety (*Vitis vinifera*) were collected after pruning at Fradelos, Vila Nova de Famalicão, Vinhos Verdes Region, Portugal. The dry material was milled on a cross-beater millSKl (Retsch, Haan, Germany), and sieved to 0.250–0.425 mm sawdust (35–60 mesh).

### 3.2. Biomass Pre-Treatment and Lignin Isolation

The biomass was submitted to a pre-treatment extraction with two different solvent mixtures (ethanol/toluene, E/T(4:1, *v*/*v*), or water/ethanol, W/E (4:1, *v*/*v*)). About 15 g of vine shoots (35–60 mesh fraction) were extracted in a Soxhlet extractor using 500 mL of a solvent mixture. The solvent was heated to the boiling point, and the extraction was performed for 6 h. The solvent was removed through rotoevaporation under vacuum, freeze-dried, and stored at −20 °C until chemical analysis. The pre-treated biomass was left to dry at room temperature overnight for further isolation of lignin by mild acidolysis, following the procedure previously described in the literature with some modifications [53]. The pre-treated dried vine shoots were placed in contact with the dioxane/water (9:1, *v*/*v*) mixture containing HCl, as described in Section S1 from the Supplementary Material. Lignin samples after precipitation, washing, and lyophilization were submitted to dialysis (membrane dialysis cutoff of 12–14 kDa, Spectrum Labs) for 24 h. Samples were coded as LET—lignin sample pre-treated with ethanol/toluene; LETD—lignin sample pre-treated with ethanol/toluene and dialyzed; LWE—lignin sample pre-treated with water/ethanol; and LWED—lignin sample pre-treated with water/ethanol and dialyzed.

### 3.3. Chemical Characterization of Extracts Obtained from Biomass Pretreatment

#### 3.3.1. Total Polyphenol Content

The total polyphenol content (TPC) of the extracts obtained from the pretreatment of biomass with E/T and W/E was determined through the Folin-Ciocalteu assay described in the literature, using gallic acid as a standard [54].

#### 3.3.2. Identification of Phenolic Compounds

The low molecular weight phenolic compounds and proanthocyanidins were determined by liquid chromatography equipped with diode array detection and mass spectrometry (HPLC–DAD/MS), as previously described in the literature [55]. For that, each sample was first dissolved in water (1 g·L^−1^) and then a microscale liquid-liquid extraction was performed using an ethyl acetate:acetonitrile (2:1) mixture. The organic phase was dried in a Labconco CentriVac Concentrator for 3 h at 37 °C. Samples were dissolved in water:methanol (1:1, *v*/*v*) and analyzed by HPLC-DAD/MS in the negative ion mode. Detailed information about the mass spectrometry (MS) equipment and experimental conditions are described in Supplementary Materials (Section S2—Table S4).

#### 3.3.3. Sugar Content

The sugar content of both samples was quantified by their derivatization with hexamethyldisilazane (HMDS) and through analysis by gas chromatography–mass spectrometry (GC-MS/MS), as described in the literature [56]. The full description of the GC–MS/MS analysis is presented in Supplementary Materials (Section S2). Glucose was used as a standard, and sugar content was expressed as mg of glucose by g of dried sample.

### 3.4. Chemical Characterization of Lignin Samples

#### 3.4.1. Lignin Content

The amount of lignin Klason was calculated in the biomass after sulfuric acid hydrolysis according to the method described in the literature [27] and fully described in Section S2 of Supplementary Materials.

#### 3.4.2. Inorganic Material, Carbohydrate, and Proanthocyanidin Content

The inorganic material was quantified gravimetrically after lignin sample incineration at 550 °C for 6 h. Carbohydrate content was determined by gas chromatography with a flame ionization detector GC-FID analysis after acid methanolysis, as previously described [57]. For quantification, the products were analyzed as described in the literature [58]. Proanthocyanidins (PAs) were quantified by the acid butanol assay adapted from the method described by Porter et al. [59]. Detailed information is described in Supplementary Materials (Section S2).

#### 3.4.3. ^13^C NMR and ^31^P NMR

Quantitative ^13^C NMR spectra were recorded in a Bruker AVANCE III 400 spectrometer operating at 400 MHz at a temperature of 318 K for 72 h. For that, 170 mg of dried lignin was dissolved in 500 μL DMSO-d_6_. ^13^C NMR measurements were determined by a simple 1D pulse sequence, a recycling time of 12 s, 1400 scans, and a 1D sequence with power-gated coupling using a 90° flip angle. For the ^31^P NMR analysis, LWED and LETD samples were accurately weighted (about 40 mg) and dissolved in 500 µL of a mixture of pyridine and deuterated chloroform (1.6:1, *v*/*v*) and left for a few minutes at room temperature until complete dissolution. Cholesterol (85 mg/mL, 100 µL) and chromium (III) acetylacetonate (5.6 mg/mL, 50 µL) were used as internal standards and relaxation reagents, respectively, as previously described [60]. The phosphitylation reagent (2-chloro-4,4,5,5-tetramethyl-1,3,2-dioxaphospholane) (100 µL) was added and the sample analyzed on a Bruker AVANCE III 400 spectrometer operating at 400 MHz at 298 K. The spectrum was acquired for 30 min with a relaxation time of 10 s.

### 3.5. Preparation of Lignin Nanoparticles

Lignin nanoparticles (LNPs) were prepared from lignin samples by nanoprecipitation or dialysis [19]. For the nanoprecipitation method, each lignin sample (LET, LETD, LWE, and LWED at 10 mg/mL) was dissolved in a mixture of acetone:water (9:1, *v*/*v*) and filtered through a 0.22 μm filter. The filtrate was added to Milli-Q water (1:20) under constant stirring using a micro syringe and left stirring for 3 h at room temperature. The suspension was centrifuged at 1500× *g* (10 min at 20 °C) to remove aggregates.

Regarding the dialysis method, lignin samples (2 mg/mL) were dissolved in tetrahydrofuran and introduced into a dialysis bag (flat width, 25 mm) with a 12–14 KDa cut-off (Spectra/Por ^®^ 2 Standard RC Tubing, Spectrum Labs, San Francisco, CA, USA), similar to what was described previously in the literature [61]. Membranes were left submerged in deionized water (1:60) under slow stirring for 24 h at room temperature with the dialysis water being periodically replaced. LNPs were stored at room temperature and characterized (average particle size, Z-average; polydispersity index, PDI; and the average zeta (ζ)-potential) by dynamic light scattering (DLS) and electrophoretic light scattering (ELS) using a Malvern Zetasizer Nano ZS instrument (Malvern Instruments Ltd., Worcestershire, UK). Samples were diluted 10 times with MilliQ-water and analyzed in triplicate. The stability of DLNPs over time (0, 7, 15, and 30 days) was assessed by following the changes in the average particle size, polydispersity index, and average zeta (ξ)-potential. Each experiment was performed in triplicate.

### 3.6. Antioxidant Activity

The antioxidant activity of extracts, LNPs, and lignin samples was performed using a 2,2′-diphenyl-1-picrylhydrazyl radical (DPPH) assay, a common spectrophotometric assay procedure for determining the antioxidant capacities of compounds. Commercial antioxidants such as trolox, ascorbic acid, and gallic acid were used as references. E/T and W/E extracts were dissolved in ethanol. LNPs were dispersed in water, while the reference antioxidants, including lignin samples (LET, LETD, LWE, and LWED), were dispersed in 50% (*v*/*v*) of ethanol, in a range of concentrations between 0 and 1 mg/mL. The full description of the DPPH assay is presented in Section S2 of Supplementary Information.

### 3.7. Statistical Analysis

All data were reported as the mean standard deviation of at least three values. Statistical analyses were performed using GraphPad Prism 7 for Windows (GraphPad Software, version 7.04, San Diego, California; www.graphpad.com). Results were subjected to an analysis of variance (one-way or two-way ANOVA), and the differences between means were calculated using Tukey’s and Holm-Sidak multiple comparison tests. They were considered significant when the *p*-values were below 0.05 (*p* < 0.001 and *p* < 0.0001).

## 4. Conclusions

This work highlights a strategy to contribute to sustainability and a circular economy with the reuse of a very abundant by-product resulting from one of the most important industries in the food sector. Furthermore, it was possible to obtain purified lignin using a simple procedure, which is increasingly used in different areas. The lignin obtained after a pre-treatment of the biomass with water/ethanol and purified by dialysis (LWED) showed a lower content of contaminants (around 9%), mainly carbohydrates (5%), and tannins (3%), when compared with the sample pre-treated with ethanol/toluene and purified by dialysis (LETD), which contains ~14% (10% of tannins and 4% of carbohydrates). The results obtained in the chemical composition of the lignins were consistent with the results obtained in the characterization of the extracts resulting from the Soxhlet procedure since the lignin treated with water/ethanol showed a lower percentage of impurities such as proanthocyanidins (tannins) and the respective extract showed a higher phenolic content when compared with the extract resulting from the Soxhlet with ethanol/toluene. Furthermore, the extract W/E showed a high content of sugar, which could be converted into lactic acid, xylitol, bioethanol, and biosurfactants.

As to the structure, the results were similar for all lignin samples, indicating that the pre-treatment and purification methodologies do not lead to modifications in lignin chemical composition. The lignin obtained showed a predominance of S over G units, with an S/G ratio between 1.45 and 1.55 and a high content of β-*O*-4 linkages. The content of phenolic hydroxyl groups was similar for both lignin samples analyzed, being 1.01 and 0.84 mmol/ g lignin for E/T dialysis and W/E dialysis, respectively. Nanoparticles proved to be very promising, with a very satisfactory size and polydispersion index. Additionally, it was very stable over time, and the antioxidant activity was higher than the ones typically reported for the commercial antioxidants BHT and BHA.

The data obtained in this study is an important tool for the design of processes to transform these agro-industrial waste materials into lignin-based high-added-value products and the production of bioactive compound extracts.

## Figures and Tables

**Table 1:** Contaminant content (ashes, carbohydrates, and tannins-proanthocyanidins) in isolated lignins (dry weight). LET (lignin pretreated with ethanol/toluene); LETD (lignin pretreated with ethanol/toluene and purified by dialysis); LWE (lignin pretreated with water/ethanol); and LWED (lignin pretreated with water/ethanol and purified by dialysis).

Lignin	Ashes, %w/w _lignin_	Carbohydrates, %w/w _lignin_	Proanthocyanidins, % w/w _lignin_
LET	0.12	5.4	11.3 ± 0.02
LETD	0.19	4.2	9.95 ± 0.03
LWE	0.30	6.9	4.65 ± 0.01
LWED	1.17	5.1	3.38 ± 0.01

**Table 2:** Assignments and quantification of the structure/linkages and functional groups identified by ^13^C NMR in the different lignin samples.

Assignments (Spectroscopic Range)	Amount (Number per Ar)			
	LET	LETD	LWE	LWED
C_β_ in β-5 and β-β structures (δ 51.0–53.8 ppm)	0.11	0.12	0.14	0.12
Aromatic OCH_3_ (δ 54.3–57.3 ppm)	1.29	1.21	1.50	1.43
Cγ in β-*O*-4 structures without Cα=O (δ 59.3–60.8 ppm)	0.39	0.36	0.42	0.39
Cγ in β-5, β-*O*-4 structures with Cα=O and β-1 (δ 62.5–63.8 ppm)	0.12	0.11	0.13	0.12
Cα in β-*O*-4 structures; Cγ in pinoresinol/syringaresinol and β-β structures (δ 70.0–76.0 ppm)	0.82	0.77	0.79	0.76
C_β_ in β-*O*-4 structures; Cα in β-5 and β-β structures (δ 80.0–90.0 ppm)	0.71	0.69	0.77	0.77
Aromatic C_Ar_-H (δ 103.0–123.0 ppm)	2.28	2.25	2.16	2.16
Aromatic C_Ar_-C (δ 123.0–137.0 ppm)	1.19	1.30	1.27	1.31
Aromatic C_Ar_-O (δ 137.0–156.0 ppm)	2.42	2.37	1.47	2.44
C_4_ in H units (δ 157.0–162.0 ppm)	0.11	0.08	0.10	0.09
CHO in benzaldehyde structures (δ 191.0–192.0 ppm)	0.02	0.02	0.03	0.02
CHO in cinnamaldehyde structures (δ 193.5–194.5 ppm)	0.02	0.03	0.04	0.03
CO in aldehydes and ketones (δ 195.0–210.0 ppm)	0.39	0.65	0.50	0.38

## Data Availability

Not applicable.

## Supplementary Materials

The supporting information can be downloaded at: https://www.mdpi.com/article/10.3390/ijms24065165/s1.
