# Supplementary material for: Exploring the Potential of Vine Shoots as a Source of Valuable Extracts and Stable Lignin Nanoparticles for Multiple Applications
Source: Int J Mol Sci. 2023 Mar 8;24(6):5165. doi: 10.3390/ijms24065165 (PMC10049713; doi:10.3390/ijms24065165)
Supplement: Supplementary file 1 [file ijms-24-05165-s001.zip › ijms-2236623-supplementary.pdf]

Supplementary Material

Exploring the Potential of Vine Shoots as a Source of Valuable Extracts and Stable Lignin Nanoparticles for Multiple Applications

Ana Rita Pereira [1], Carina Costa [2,3,*], Nuno Mateus [1], Victor de Freitas [1], Alírio Rodrigues [2,3] and Joana Oliveira [1,*]

[1] Laboratório Associado para a Química Verde–REQUIMTE, Departamento de Química e Bioquímica, Faculdade de Ciências, Universidade do Porto, Rua do Campo Alegre, 687, 4169-007 Porto, Portugal
[2] LSRE-LCM-Laboratory of Separation and Reaction Engineering-Laboratory of Catalysis and Materials, Faculty of Engineering, University of Porto, Rua Dr. Roberto Frias, 4200-465 Porto, Portugal
[3] ALiCE-Associate Laboratory in Chemical Engineering, Faculty of Engineering, University of Porto, Rua Dr. Roberto Frias, 4200-465 Porto, Portugal
* Correspondence: caecosta@fe.up.pt (C.C.); jsoliveira@fc.up.pt (J.O.)

Section 1: Results

Table S1 - Molecular weight and evaluation of the polydispersity of the different lignin fractions. LET – lignin sample pre-treated with ethanol/toluene; LETD – lignin sample pre-treated with ethanol/toluene and dialyzed; LWE – lignin sample pre-treated with water/ethanol and LWED – lignin sample pre-treated with water/ethanol and dialyzed.

Lignin	Molecular weight ($g.mol^{-1}$)	Polydispersity
**LET**	45,243	1.43
**LETD**	48,249	1.42
**LWE**	43,340	1.44
**LWED**	47,254	1.40

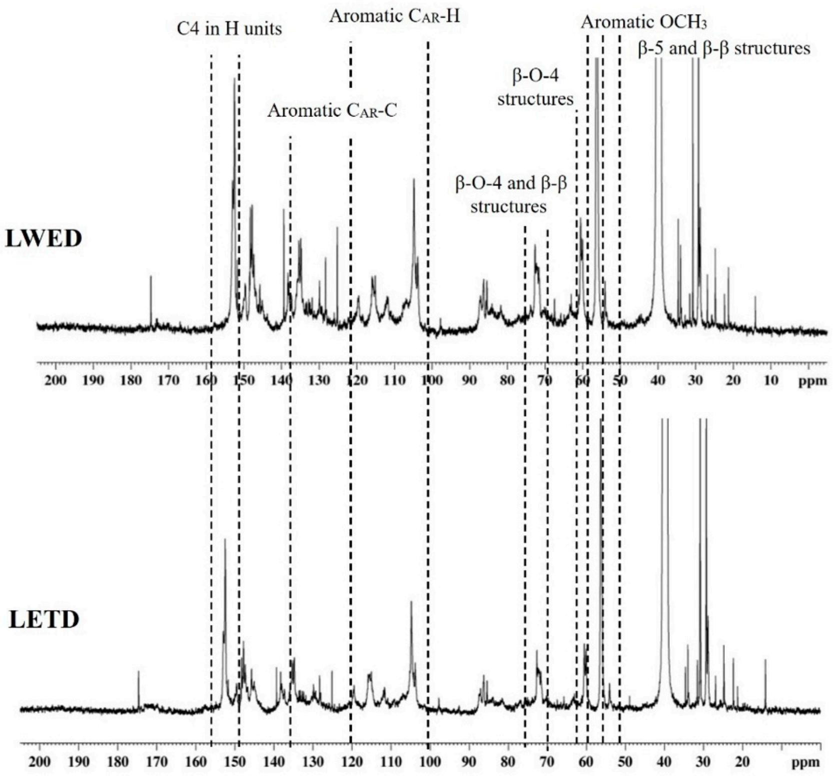

Figure S1 - Quantitative ^{13}C NMR spectra of lignin extracts from vine shoots: LETD – lignin pre-treated with ethanol/toluene and dialyzed and LWED – lignin pre-treated with water/ethanol and dialyzed.

Table S2 - β-*O*-4 structures content (number per 100 aromatic rings), degree condensation (DC), S:G:H and S/G ratio determined for the lignin samples.

	**β-*O*-4 structures**	**DC, %**	**S:G:H**	**S/G**
**LET**	60	9	54:36:10	1.48
**LETD**	57	13	54:38:08	1.45
**LWE**	63	19	55:35:10	1.55
**LWED**	65	21	54:37:09	1.47

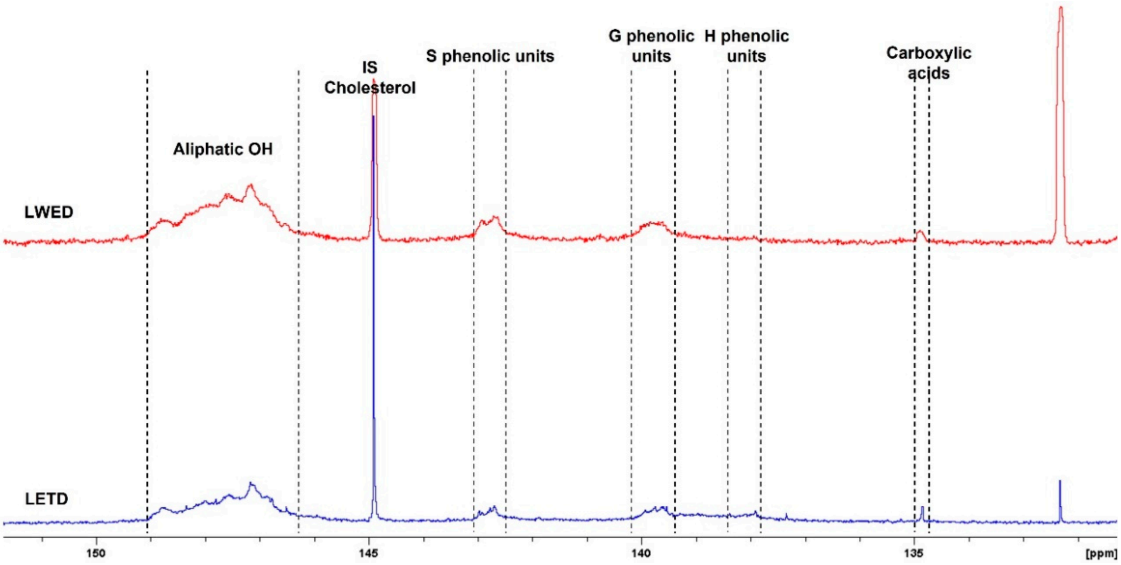

Figure S2 - Quantitative ^{31}P NMR spectra (δ 130-150 ppm) for phosphitylated lignins LETD and LWED.

Table S3 - Quantification of phenolic and aliphatic hydroxyl groups, and carboxylic acids in lignins LETD and LWED by ^{31}P NMR.

Assignments	Amounts (mmol/g $_{lignin}$)	
	**LETD**	**LWED**
Aliphatic OH	3.20	2.80
Carboxylic acids	0.05	0.07
**Total phenolic units**	1.01	0.84
Non-condensed phenolic units		
S phenolic units	0.30	0.37
G phenolic units	0.48	0.41
H phenolic units	0.23	0.06

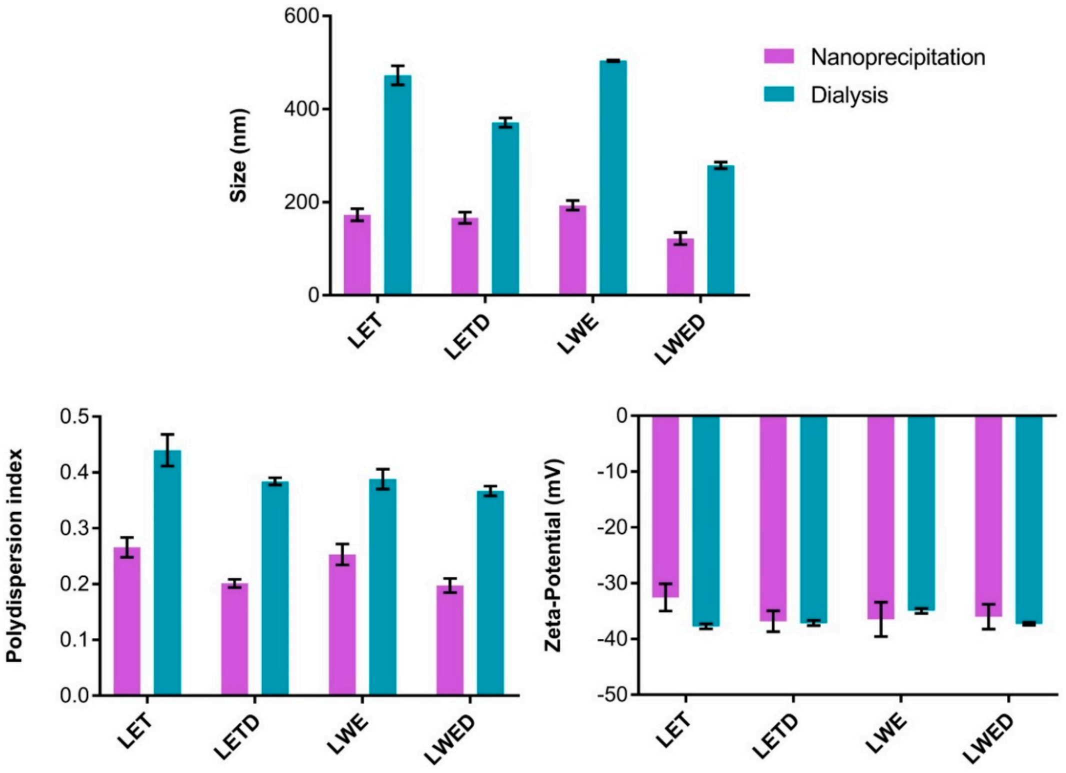

Figure S3 - Evaluation of physical properties (size, polydisperse index, and zeta-potential) of LNPs prepared by nanoprecipitation or dialysis method.

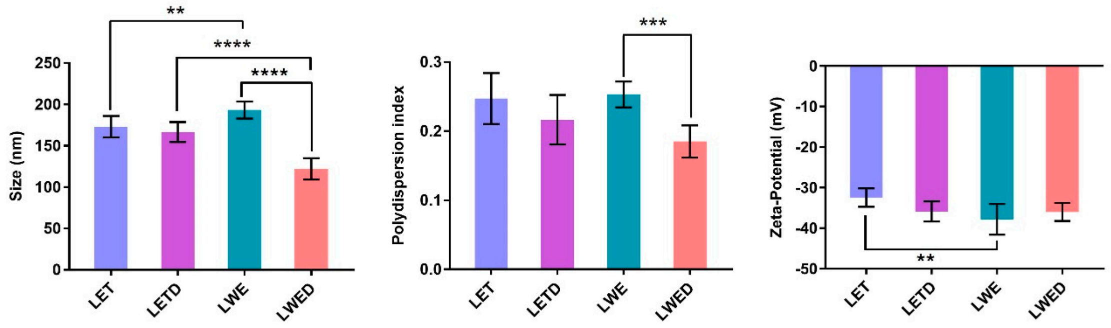

Figure S4 - Comparison of physical properties of lignin nanoparticles obtained by nanoprecipitation for different isolated lignins. Statistical analysis was made by ANOVA, followed by a Turkey's test. Different asterisks indicate a statistical difference: $p < 0.005$ (**), $p < 0.001$ (***) and $p < 0.0001$ (****). Errors bars represent the mean ± s.d (n=9).

Section 2: Materials and methods

Lignin isolation and purification

The pre-treated dried vine shoots were placed in contact with the dioxane/water (9:1, v/v) mixture containing HCl 0.2 M, under nitrogen and reflux for 40 minutes. This procedure was performed two more times but in the last extraction, the HCl was not added to the dioxane/water mixture. Lignin was precipitated in cold water after concentration of the previous fractions by evaporation under vacuum. The precipitated lignin was removed by centrifugation, washed with water until neutral pH, and freeze-dried. Lignin samples were submitted to dialysis (membrane dialysis cutoff 12-14 kDa, Spectrum Labs) at a concentration of 2 mg/mL for 24 h, replacing the water several times. Samples were coded as: LET – lignin sample pre-treated with ethanol/toluene; LETD – lignin sample pre-treated with ethanol/toluene and dialyzed; LWE – lignin sample pre-treated with water/ethanol and LWED – lignin sample pre-treated with water/ethanol and dialyzed.

Determination of low molecular weight phenolic compounds and proanthocyanidins

Mass Spectrometry analysis was performed using a Finnigan Surveyor series liquid chromatograph, equipped with a reversed-phase C18 column (LIchrospher® 100) with 250 × 4.6 mm i.d., 5 µm thermostatted at 25 °C. The mass detection was carried out in the negative ion mode in a Finnigan LCQ DECA XP MAX (Finnigan Corp., San José, CA, USA) mass detector with an API (Atmospheric Pressure Ionization) source of ionization and an ESI (ElectroSpray Ionization) interface. Spectra were recorded in the negative ion mode between m/z 300 and 1500. The elution procedure was presented in **Table S4**.

Table S4 - Elution conditions for the identification of low molecular weight phenolic compounds and proanthocyanidins by LC-MS analysis.

Time (minutes)	Flow mL/min	Solvent A: 1% acetic acid in water	Solvent B: 1% acetic acid and 20% acetonitrile in water
0	0.3	80	20
55	0.3	20	80
70	0.3	10	90
90	0.3	0	100
100	0.3	0	100
105	0.3	80	20
115	0.3	80	20

Sugar content – GC-MS/MS conditions

GC-MS/MS was carried out with a Trace 1300 gas chromatograph equipped with a split–splitless injector, an autosampler 1310 Thermo Scientific, and an ISQ Single quadrupole MS (Thermo Fisher, Austin, TX, USA). A total of 1 μL of the sample was injected into the injector operating in splitless mode. The temperatures of the injector and the MS-transfer line were 250°C and 300°C, respectively. Compounds were separated on a 30 m × 0.25 mm (i.d.) × 0.25 μm DB-17 capillary column (Agilent Technologies, CA, USA) operating at a constant helium flow of 1.5 $mL.min^{-1}$. The column temperature was initially set to 110 °C, held for 5 min, increasing then at a rate of 6$°C.min^{-1}$ to 300°C and held for 5 min. Measurements were performed in SCAN mode with the *m/z* range set to 40–1100. The MS conditions were as follows: ion source temperature 280°C and electron energy 70 eV, using glucose as standard for the calibration curve. Selected ion monitoring (SIM) conditions were used for the glucose, selecting the *m/z* 204.

Lignin content – Klason lignin

A solution of 72 % (w/w) H_2SO_4 was added to 1.0 g of residue and after 2.5 h the mixture was diluted with water and hydrolyzed for 2 h under reflux. Then, the final material was filtrated, washed with water until neutral pH, dried, and weighed. For the quantification of acid-soluble lignin, 20 mL of the filtrate was adjusted to pH 9 and mixed with 50 mg of $NaBH_4$ and left stirring for 20 min. Following this, the solution was acidified, diluted, and the absorbance at 280 nm was measured using a solution of 1.8% H_2SO_4 as a reference. This estimate was adjusted for moisture content.

Carbohydrates and proanthocyanidins content in lignin samples

For the carbohydrates content determination, 15 mg of material was dissolved in 2 mL of a methanol solution containing 2 M HCl and submitted to acid methanolysis at 100 °C for 4 h. After cooling, pyridine and sorbitol (internal standard solution) was added, and the mixture was evaporated under reduced pressure. The dried methanol derivatives were converted into trimethylsilylated. For quantification, the products were analyzed by gas chromatography with a flame ionization detector (GC-FID), as already described in the literature (1).

Regarding the proanthocyanidins content, into pressure and temperature-resistant tubes, 500 μL of butanol reagent (butanol with 5% (v/v) of concentrated hydrochloric acid) were mixed with 18 μL of ammonium iron (III) sulfate dodecahydrate solution (20 $mg.mL^{-1}$ prepared in aqueous hydrochloric acid (2 M)) and 82.5 μL of a methanolic solution of each lignin fraction (3.0 mg/mL: LET, LETD, LWE, and LWED). The mixture was incubated at 100°C for 50 min, and after cooling, the absorbance was determined

at 520 nm was determined. The quantification was made using as standard a purified PACs fraction containing trimers and based on the calibration curve (y = 1.7091x + 0.0519, r^2 = 0.9992).

Gel permeation chromatography (GPC)

The molecular weight of lignin samples was determined on a Shimadzu UFLC with a diode array (running at 280 nm wavelength) and refraction index detectors. Two Agilent gel columns (OligoPore column 300×7.5 mm and a MesoPore column 300×7.5 mm) placed in series with a guard column Oligopore 50×7.5 mm were used. GPC analysis was performed at a flow rate of 0.8 $mL \cdot min^{-1}$ using an isocratic gradient and dimethylformamide with 0.5% w/v of lithium chloride as the mobile phase. The temperature of the columns was maintained at 70°C during the acquisition. Polystyrene standards with molecular weights ranging from 162 to 55,000 $g \cdot mol^{-1}$ and measured at 268 nm were used to calibrate the equipment prior to sample analysis. Solutions (5 $mg \cdot mL^{-1}$) of each polystyrene standard and each lignin sample were dissolved in dimethylformamide with 0.5% w/v of lithium chloride, stirred, and filtered (0.2 μm) before injection.

FTIR

FTIR spectra of lignin samples were recorded on a Spectrum Two FT-IR Spectrometer (PerkinElmer). Potassium bromide (KBr) discs of each sample were prepared by pressing a mixture of 100 mg of KBr and 1 mg of sample under vacuum. The spectra were recorded in the range of 4000-400 cm^{-1} with 4 scans in the medium-infrared area.

DPPH assay

For the DPPH assay, in 96-well plates, 270 μL of DPPH solution (prepared in methanol at a concentration of 24.2 $\mu g.mL^{-1}$) was mixed with 30 μL of antioxidant samples. For each sample concentration, the radical scavenging reaction was monitored as a function of time by measuring the DPPH absorbance band at 517 nm (λ_{max}) on a plate reader (Biotek Powerwave XS), in the beginning, and after 30 min of reaction. IC_{50} values of test/reference compounds are represented as half of the maximum inhibitory concentration of DPPH radical scavenging activity. The antioxidant activity was expressed as the radical scavenging activity (RSA) by calculating the fitting peak area at 517 nm, using the following equation:

$$(RSA\%) = \frac{(Abs\ control - Abs\ sample)}{Abs\ control} \times 100$$

where Abs control and Abs sample are the absorbance of the control at t = 0 min and the tested sample at different incubation times, respectively.

1. Costa C, Alves A, Pinto PR, Sousa RA, Borges da Silva EA, Reis RL, et al. Characterization of ulvan extracts to assess the effect of different steps in the extraction procedure. Carbohydrate Polymers. 2012;88(2):537-46.
